# News and Notes

**Published:** 1910-06

**Authors:** 


### NEWS AND NOTES

### PROGRAM OF THE CHATTAHOOCHEE VALLEY MEDICAL ASSOCIATION TO BE HELD IN ROANOKE, ALA., JULY 12-13.

### FIRST DAY.

Call to order 12:00 M.

Invocation—By Rev. F. H. Farington.

Address of Welcome on Behalf of the City of Roanoke—By Mayor F. P. Nichols.

Address of Welcome on Behalf of Randolph Medical Society—By Dr. W. G. Floyd, Roanoke, Ala.

Response on Behalf of Chattahoochee Valley Medical and Surgical Association—By Dr. J. G. Palmer, Opelika, Ala.

#### AFTERNOON SESSION.

Diabetes—By C. S. Yarbrough, M. D., Auburn Ala. Leaders in discussion: J. H. McDuffie, T. M. Sprewell, L. W. Johnston.

Pellagra—By Thos. F. Taylor, M. D., Tuskegee, Ala. Leaders in discussion: Jas. S. McLester, O. S. Justice, Jas. T. Rushin.

Puerperal Infection—By J. T. Rushin, M. D., Tallassee, Ala. Leaders in discussion: R. S. Hill, A. B. Bennett, C. L. Williams.

Report of Case of Pellagra—By H. L. McLendon, M. D., West Point, Ga. Leaders in discussion: Thos. F. Taylor, J. P. Watkins, J. M. Poer.

Report of Case of Pellagra—By A. D. McLain, M. D., Salem, Ala. Leaders in discussion: J. H. McDuffie, H. L. McLendon, W. P. Dickinson.

Correction of Retro-Displacements of the Uterus—By J. D. S. Davis, M. D., Birmingham, Ala. Leaders in discussion: R. S. Hill, Gaston Torrence, W. L. Cooke.

Intestinal Indigestion with Reference to Diarrhœa—By Dr. Saliba, Roanoke, Ala. Leaders in discussion: Geo. M. Niles, J. L. Campbell, C. A. Dexter.

EVENING SESSION 8:30 P. M.

The Economic and Sociologic Value of Preventive Medicine —By B. F. Rea, LaFayette, Ala. Leaders in discussion: Jas. J. Win, Geo. M. Niles, F. M. Ridley, Jr.

A Consideration of Some of the Modern Methods of Home Sanitation—By C. A. Cary, D. V. S., Auburn, Ala. Leaders in discussion: Jas. S. McLester, Hugh M. Lokey, H. S. Slack.

The Physician's Duty Towards Educating the Public on Hygenic and Sanitary Subjects—By C. A. Dexter, M. D., Columbus, Ga. Leaders in discussion: C. A. Cary, Jas. J. Win, H. B. Disharoon, J. M. Welch.

---

### SECOND DAY.

MORNING SESSION 8:00 A. M.

Significance of Hæmatemesis—By Geo. M. Niles, M. D., Atlanta, Ga. Leaders in discussion: H. M. Terrell, A. L. Harlan, H. P. Hamner.

Some of the Newer Methods of Diagnosis of Intestinal Diseases—By L. M. Gaines, M. D., Atlanta, Ga. Leaders in discussion: Dr. Saliba, D. Y. Sage.

Chronic Non-Gonorrhœal Infection of the Prostate Gland—By E. G. Ballenger, M. D., Atlanta, Ga. Leaders in discussion: J. A. Goggans, W. L. Cooke, Hugh McCulloh.

Typhoid Fever—By J. M. Poer, M. D., West Point, Ga. Leaders in discussion: W. S. Elkin, W. G. Floyd, J. P. Motley.

Report of Two Cases: (1) Removal of Complete Shaft of Tibia, Replaced by Transplantation of Fibula. (2) Report of Cases of Extreme Prolapse of Uterus, Operated on by Schautas Method—By Gaston Torrence, M. D., Birmingham, Ala. Leaders in discussion: F. M. Ridley, Sr., R. S. Hill, J. D. S. Davis, H. S. Bruce.

Some Surgical Manifestations in Hysteria—By Wesley Taylor, M. D., Atlanta, Ga. Leaders in discussion: H. B. Disharoon, Velpeau Langley, J. G. Palmer.

The Development and Nerve Supply of the Intestinal Canal Surgically Considered—By J. L. Campbell, M. D., Atlanta, Ga. Leaders in discussion: Thos. F. Taylor, Geo. M. Niles, W. T. Langley.

Acute Nephritis—By J. H. McDuffie, M. D., Columbus, Ga. Leaders in discussion: C. S. Yarbrough, Auburn; H. S. Bruce, S. H. Newman, W. W. Stevenson.

Headaches and Neuralgias from Intra-Nasal Pressure and Accessory Sinus Diseases—By Hugh M. Lokey, M. D., Atlanta, Ga. Leaders in discussion: H. S. Persons, T. E. Mitchell, Geo. W. Chambers.

Chronic Appendicitis, with Report of Cases—By W. L. Cooke, M. D., Columbus, Ga. Leaders in discussion: L. L. Hill, F. M. Ridley, Sr., E. P. Greene.

---

AFTERNOON SESSION 2:00 P. M.

Labor and Its Proper Management—By A. B. Bennett, M. D., Opelika, Ala. Leaders in discussion: W. W. Stevenson, C. A. Dexter, John T. Striplin.

Some Experiences in Minor Medicine—By P. M. Lightfoot, M. D., Cross Keys, Ala. Leaders in discussion: W. L. Johnston, Thos. F. Taylor, T. W. Haralson.

Paresis—By Stewart Roberts, M. D., Atlanta, Ga. Leaders in discussion: J. A. Goggans, H. L. McLendon, Waverly, Ala., C. A. Dexter.

The Pathology and Treatment of Ununited Fractures—By J. N. Baker, M. D., Montgomery, Ala. Leaders in discussion: J. P. Watkins, H. M. Terrell, F. M. Ridley, Jr., W. D. Gaines.

Considerations in Surgical Convalescence—By Herbert P. Cole, M. D., Mobile, Ala. Leaders in discussion: F. M. Ridley, Sr., W. L. Cooke, A. D. McLain.

Chronic Non-Suppurating Diseases of the Middle Ear—By Geo. H. Cooper, M. D., Opelika, Ala. Leaders in discussion: W. L. Bullard, T. E. Mitchell, Martin Crook.

Burns—Homer Boatright, M. D., Carrollton, Ga. Leaders in discussion: J. M. Welch, W. L. Elkin, C. A. Dexter.

Some Experiments with Tuberculin—By W. P. Dickinson, M. D., LaFayette, Ala. Leaders in discussion: L. M. Gaines, W. T. Langley, C. A. Dexter.

Variations in Appendicitis—Dr. F. K. Bolarld, Atlanta, Ga.

### REGULAR MEETING OF THE FULTON COUNTY MEDICAL SOCIETY, APRIL 28, 1910.

REPORTED BY R. R. DALY, M. D.

Dr. Robin Adair read a paper on "Prophylaxis and the Dental Nurse."

Dr. Amster said that the subject was timely and important. Incomplete hygiene of the mouth is the cause of imperfect metabolism throughout the body. The drying of the gastric secretions was frequently due to the improper condition of the food coming through a septic mouth. Bad breath is due to bad teeth rather than to bad stomach. Often arterio sclerosis begins with the improper digestion at the mouth. Prophylaxis should be extended to the tongue and tonsils.

Dr. Daly said that the subject of the dental nurse had not been touched upon by the essayist although it formed a part of the title of his paper. He called attention to the fact that the high-class dentist was in close touch with the best physicians in trying to prevent disease and in doing so he was rendering a service which tended to make his own income less. Not only that, but the price he received for this service was far less than he really earned. It takes more time properly to clean the patient's teeth than the majority of the patients are willing to pay for. This matter has to be met in some way or the dentist could not prosper. It has been found impracticable to bring in a graduate dentist assistant because that man soon wishes to do more important work. Dr. Adair has instructed a lady in the proper technique of cleansing the teeth and now has her as an efficient assistant in his office. This lays him open to some criticism perhaps on account of its novelty, but that criticism is not sustained. Dr. Daly, himself, has been under her care and finds her efficient. In his own practice he has occasion frequently to send patients to the dentist before he can treat their throats and many cases of tubercular pharingitis cannot be helped until their teeth are properly cleansed. He advocates the instruction of teeth cleaning in high class training schools for nurses.

Dr. Toepel said prophylaxis should be begun in the schools.

Our teachers are taught to explain this theoretically and the use of the tooth-brush has increased materially. Five years ago at the State Medical meeting this matter was introduced by him and suitable resolutions were adopted. He believes that dentists should be appointed to care for the schools.

Dr. Lindorme said that his own child was told by the school examiner to have its mouth attended to. He said several cavities were found in the cleaning, the remedying of which increased his child's health greatly. His children all now take pride in their teeth.

Dr. Visanska said that he always instructs mothers to cleanse their children's teeth and in his clinics in the mill district he has what is called "The Tooth Brush Brigade" for the education of children and adults. We should all support Dr. Roberts in his excellent school work.

Dr. Champion said that he enjoyed the paper greatly and that the principles applied to adults as well as to children. In his own practice he finds that syphilitics frequently have bad teeth and they can not be treated until their mouths are in proper condition.

Dr. Adair said, in closing, that it was rarely that even cultivated people knew how to use a tooth-brush properly. The ordinary way of simply brushing across the teeth was fallacious but it was the usual custom. The teeth should be brushed from the gums toward the edges. At seven years of age children start in new teeth and all of these should be in good condition. In order to make them so the earlier teeth should have had the best care so that they would stay in place until pushed off by the properly developing new ones. In surgery possibly one-half the autointoxication with which the patients suffer comes from the mouth. In New York City the dental nurse is legalized and she should be trained and legalized here. As it is even the best skilled nurses do not know how to cleanse the teeth of patients in the hospitals and Dr. Adair has seen cases returning from short febrile attacks in hospitals with mouths in such bad order that it took many days to restore them.

In discussing the Cause of Constipation, Dr. Toepel said that irregular hours, inconvenient closets, drug habit, enemas.

etc., interfered with the normal physiological action. Obstructive and surgical cases should be carefully investigated. The treatment implies, first, prophylaxis, then proper feeding, after which comes the use of drugs, exercise, hydrotherapy, massage and vibratory applications.

Dr. Duncan said that his first principle was to avoid purgatives; that they tended to produce constipation. Vibratory massage and hydrotherapy are good things but regular habits and proper digestion will render them unnecessary.

Dr. Amster said that several years ago he condemned the use of drugs in constipation. The spastic and atonic forms should be diagnosed and the treatment shaped to what is found. Vegetables and fruits, well chewed, will cure almost any case of constipation if aided by warm oil injections.

Dr. Visanska said in school children have to leave home earlier than they should and after arriving at school frequently have difficulty in convincing the teacher that they need to leave the room.

---

### REGULAR MEETING OF THE FULTON COUNTY MEDICAL SOCIETY, THURSDAY, MAY 5, 1910.

REPORTED BY R. R. DALY, M. D.

Dr. R. R. Kime read a paper on "After Treatment in Pelvic and Abdominal Surgery."

Dr. Crowe said that his own rule through all abdominal surgery is to maintain simplicity of technique and operation. Often he has to do incomplete operations because the condition of the patient will not permit him to make extensive investigations and removals of other organs that may be slightly diseased. During the past three years he has given 10 drops of adrenaline chloride repeated in six hours after the operation, then he has used proctoclysis of black coffee 2 oz., normal salt 6 oz. The use of strychnia immediately after operation is not good practice. Codein for pain is p. r. n. but is to be avoided as much as possible. He finds it is better to change the dressings in thirty-six hours to relieve the patient of the drag of them rather than to wait four or five days. As to position, he elevates the head whenever there is extensive proctoclysis in order to prevent flooding of the endocardium. He raises the head from a few inches to Fowler's position. On the fourth or fifth day he introduces the treatment of iron, quinine and strychnine.

Dr. McRae said that in the after treatment it was of great importance to let the patients alone and permit them to be comfortable as much as possible. Even if the bowels do not move for two to four days it is of no importance if the condition of the patient remains good. Purgatives disturb the patient and are likely to cause a rise in temperature. In septic cases and where there are large amounts of raw surfaces likely to form adhesions, he promptly used eserine and strychnine and places either in the Fowler's or Park's position so as to have the abdominal viscera draw away from the exposed surfaces. The Fowler's position and Murphy proctoclysis are invaluable in pelvic disturbances, but McRae does not try to follow Murphy's details because he thinks it is unnecessary. He likes to use forceps on the tube to regulate the flow. Saline enemas should intermit with the proctoclysis after the first twenty-four hours.

Dr. Barnett said that if any part of the proctoclysis tube was below the level of the patient, air collects in the loop and interferes with the operation. He has not found adrenalin of value in his practice.

Dr. Kime in closing said that he has used adrenalin when he has feared capillary hemorrhages. As to proctoclysis, it can be abused as can anything else, but when it is indicated there is nothing so good. It is hard to get a nurse to use it properly and make the injection sufficiently slow and steady. A special apparatus is advisable because the fluid must be kept warm. Any constriction on the tube is likely to prevent the return flow of gas.

Dr. King read a paper on "Some Diagnostic Points of Mental Diseases," which is found in another part of the JOURNAL.

Dr. J. B. Baird said that the difficulty of diagnosis in insanity is in finding the normal standard. It must be judged by the condition of each man's normal mind. Thoughts are manifested by speech and action and men can not think alike no matter how they try. The general average is uncertain. For these reasons it is advisable that there should be a jury who may be able to look upon all sides of a man's condition before repriving him of his liberty. The diagnosis is likely to be uncertain if left to two physicians and a man should not be deprived of his liberty for several months on their statement. When one is committed to an asylum in this state it is for a definite minimum time.

Dr. Daly said that he was greatly interested in this subject, having had several years' experience in the care of the insane. It is true that there is no definition of insanity and that the nearest approximation to one would be a more or less prolonged departure from the patient's usual way of thinking, feeling and acting. This standard in each individual was fairly easily ascertained unless the mental difficulty had been lasting over a period of several years. So far as the general average of mentality is concerned, there is a standard and scientific men in the psychologic laboratories of the world are making this clearer every day. It is amply sufficient to have a man examined by two competent physicians and their findings approved by a Judge of a Court of Record. This protects the patient's liberty and saves him from the disagreeable conditions imposed by appearing before a jury of men who know nothing of insanity. It is a clear outrage to place an insane man, or woman, in jail while waiting for the next ten days' period to pass before the patient may be sent to the hospital. The people fail to recognize that insanity is a disease just as much as typhoid fever or pneumonia and that it should have proper treatment as quickly as either of these well-known diseases. So far as illegal incarceration is concerned, every patient is a ward of the court and so long as the findings of the doctors are passed upon by a Justice of the Court of Record there can be no chicanery. More than this, the doctors in charge of the asylums are reputable men and they, too, would prevent the admission of the non-insane. As the essayist has said, our present laws should be radically changed and if necessary a State Commission should be appointed to supervise all of these matters.

Dr. Clarke said that Georgia was behind the age in many ways. In the matter of the age of consent in this state which is from 10 to 12 years and only last year were criminals relieved from practical slavery. The essayist's paper should be brought to public attention and the intent should be carried out legally. Juries are usually made up of "hangers-on" any way and the worst of the entire procedure is the jail incarceration. He does not believe, however, that two men are enough to examine a case.

Dr. Kime said that several years ago he laid this matter before the State Society. He believes that the Superintendent of an asylum should have power to dismiss a case whenever he thinks proper. He agrees that the composition of the jury is usually of a low order.

Dr. Toepel said that it was far easier under the present management to send patients away improperly than it would be under the two physicians' certificate. Juries are made up of inefficient men and usually the physician is inattentive. He has heard questions asked which were entirely irrelevant and has seen patients committed without any adequate evidence as to their mental condition. The State Commission, if appointed, should have supervision of methods to improve the condition of backward children in the schools.

Dr. Archibald Smith said that he had the case of a woman who was delivered only six months previously. She was insane and committed to the jail to await examination.. The proceedings before the jury were careless and not particularly indicative of insanity.

Dr. King said that our classification of insanity is growing so definite and our scientific research so accurate that we can diagnose insanity. Georgia is one of the six states so far behind that it requires a jury to establish commitment. He has at his private sanatorium almost constantly applications from poor people to allow their patients to remain under his care during the ten days rather than go to jail.

